# Relationship of Circulating Hyaluronic Acid Levels to Disease Control in Asthma and Asthmatic Pregnancy

**DOI:** 10.1371/journal.pone.0094678

**Published:** 2014-04-15

**Authors:** Noémi Eszes, Gergely Toldi, Anikó Bohács, István Ivancsó, Veronika Müller, János Rigó Jr., György Losonczy, Barna Vásárhelyi, Lilla Tamási

**Affiliations:** 1 Department of Pulmonology, Semmelweis University, Budapest, Hungary; 2 1st Department of Pediatrics, Semmelweis University, Budapest, Hungary; 3 1st Department of Obstetrics and Gynecology, Semmelweis University, Budapest, Hungary; 4 Department of Laboratory Medicine, Semmelweis University, Budapest, Hungary; 5 Research Group of Pediatrics and Nephrology, Hungarian Academy of Sciences, Budapest, Hungary; Gentofte University Hospital, Denmark

## Abstract

Uncontrolled asthma is a risk factor for pregnancy-related complications. Hyaluronic acid (HA), a potential peripheral blood marker of tissue fibrosis in various diseases, promotes eosinophil survival and plays a role in asthmatic airway inflammation as well as in physiological processes necessary to maintain normal pregnancy; however the level of circulating HA in asthma and asthmatic pregnancy is unknown. We investigated HA levels in asthmatic patients (N = 52; asthmatic pregnant (AP) N = 16; asthmatic non-pregnant (ANP) N = 36) and tested their relationship to asthma control. Serum HA level was lower in AP than in ANP patients (27 [24.7–31.55] vs. 37.4 [30.1–66.55] ng/mL, p = 0.006); the difference attenuated to a trend after its adjustment for patients’ age (p = 0.056). HA levels and airway resistance were positively (r = 0.467, p = 0.004), HA levels and Asthma Control Test (ACT) total score inversely (r = −0.437, p = 0.01) associated in ANP patients; these relationships remained significant even after their adjustments for age. The potential value of HA in the determination of asthma control was analyzed using ROC analysis which revealed that HA values discriminate patients with ACT total score ≥20 (controlled patients) and <20 (uncontrolled patients) with a 0.826 efficacy (AUC, 95% CI: 0.69–0.97, p = 0.001) when 37.4 ng/mL is used as cut-off value in ANP group, and with 0.78 efficacy (AUC, 95% CI: 0.65–0.92, p = 0.0009) in the whole asthmatic cohort. In conclusion circulating HA might be a marker of asthma control, as it correlates with airway resistance and has good sensitivity in the detection of impaired asthma control. Decrease of HA level in pregnancy may be the consequence of pregnancy induced immune tolerance.

## Introduction

Asthma is a chronic inflammatory disease of the airways characterized by variable and recurring symptoms, local inflammation, reversible airflow obstruction, and bronchospasm [1]. Asthma is a prevalent chronic disease which is not optimally controlled in about 50% of cases even in developed countries. It has a high burden of morbidity especially if not controlled [2]; however, objective serum markers reflecting asthma control are not known.

Airway inflammation, a major element of asthma pathophysiology, has been described to be related to asthma severity [3], [4] and asthma control [5] and causing systemic signs of inflammation as well [1]. Easily obtainable markers of systemic inflammation especially those related to clinical control of asthma may help in clinical decision-making. Recently, a number of studies investigated the sensitivity of circulating inflammatory markers in the evaluation of asthma control. Increase in proinflammatory cytokines, such as interleukin-6 [6] and tumor necrosis factor-α [7], [8] have already been described in asthmatic patients as well as elevated circulating C-reactive protein levels in nonallergic [9], and neutrophilic asthma [10]. In steroid-naive asthmatic patients C-reactive protein levels negatively correlated with indices of lung function (forced expiratory volume in one second (FEV1), FEV1/forced vital capacity and forced mid-expiratory flow) and positively with sputum eosinophil count [11]. In our recent study another inflammatory biomarker, serum soluble urokinase plasminogen activator receptor (suPAR) was shown to correlate with airway resistance having a good sensitivity in the detection of impaired asthma control [12].

Asthma is one of the most frequent potentially serious medical conditions complicating pregnancy, occurring in 3.7 to 8.4 percent of pregnant women [13]. It is a risk factor for several obstetrical and fetal complications including preeclampsia, Cesarean section, preterm delivery, low birth weight and a high risk of perinatal mortality [14]–[16]. In addition, pregnancy has also an effect on asthma control deteriorating the symptoms in one-third of pregnant women [17]; therefore, monitoring and treatment of pregnant women with asthma is a particular challenge. Optimal asthma control during pregnancy decreases maternal and neonatal risks [18]; hence non-invasive assessment of asthma control and appropriate management is of particular importance. However, although some clinical factors (such as more severe asthma before pregnancy, disease worsening in previous pregnancies [15], [17], [19] and impaired asthma-specific quality of life in early pregnancy [20] may predict the changes of asthma control during pregnancy, to date systemic markers related to asthma control determinants or any lung function parameters in asthmatic pregnancy are missing.

During last decades elevated serum level of hyaluronan (hyaluronic acid; HA), a major extracellular matrix component, was reported in several pathological conditions characterized by systemic inflammation and activation of immune system (e.g. sepsis, rheumatoid arthritis); however its suitability as a systemic inflammation marker in asthma has not yet been investigated. HA is a glycosaminoglycan forming non-covalent complexes with extracellular matrix proteoglycans [21], [22]. It is a structural building molecule of many healthy tissues which degrades to low molecular weight HA during pathological conditions such as immune activation [23], systemic inflammation [24], tissue injury and repair [25], and further stimulates chemokines, cytokines, adhesion molecules, transcription- and growth factors, activate fibroblasts, epithelial- and inflammation cells thus generating proinflammatory and proangiogenic effects [26]. Normal serum concentration of HA may vary from 10 to 100 µg/L [27]. According to available data, inflammatory response leads to elevated circulating HA in many inflammatory conditions, such as liver and pulmonary fibrosis, atherosclerosis, diabetes, certain tumors, chronic obstructive pulmonary disease [28].

Recently HA was shown to be implicated in asthma pathophysiology by contributing to the two main characteristics of the disease: airway inflammation and remodeling. Elevated levels of HA have been reported in bronchoalveolar lavage fluid of persistent asthmatic patients [29], [30] and they correlated with the severity of disease [31]. A recent study has shown decreased elimination and increased responsiveness to HA in asthmatic macrophages which may lead to persistent airway inflammation [32]. In addition, HA promotes eosinophil survival in a dose-dependent manner that may play a further role in chronic asthmatic inflammation [33].

HA also plays a role in maintaining healthy pregnancy [34]. Serum HA levels increase near term, reflecting the process of cervical ripening [35]. Furthermore, two studies indicated HA to be a predictive marker of preeclampsia in the late pregnancy [36], [37]. However, despite of the known role of HA in the mechanism of asthma and pregnancy, as well as findings in bronchoalveolar lavage fluid, airway smooth muscle and endobronchial biopsy, to date data on circulating HA levels and their possible relationship to disease control are scarce either in asthma or in asthmatic pregnancy.

Therefore, present study aimed to investigate serum HA levels in patients with asthma and asthmatic pregnancy. Furthermore, in order to define the utility of HA as a screening tool in the evaluation of asthma control in asthmatic pregnant and non-pregnant patients, we also investigated the relationship between HA and asthma control determinants.

## Methods

### Ethics Statement

Written informed consent was obtained from all subjects, and our study was reviewed and approved by an independent ethical committee of the institution (Institutional and Regional Research Ethics Committee of Semmelweis Medical University). Laboratory studies and interpretations were performed on coded samples lacking personal and diagnostic identifiers. The study was adhered to the tenets of the most recent revision of the Declaration of Helsinki.

### Study Participants

The study had a cross-sectional design. 36 asthmatic non-pregnant (ANP) women and 16 asthmatic pregnant (AP) patients were enrolled. Asthmatic patients were assessed at their regular visit at the outpatient clinic of the Department of Pulmonology, Semmelweis University. They had persistent disease and asthma had been diagnosed according to the current guidelines (at least 6 months prior to the study) [1]. Exclusion criteria were diabetes mellitus, autoimmune disease, cardiovascular diseases, renal disorder, liver diseases, untreated hypertensive disorder, angiopathy, maternal or fetal infection, fetal congenital anomaly, multi-fetal gestation, current smoking or more than 5 pack years of smoking history, any other chronic disease (except for allergic rhinitis), and acute infection within four weeks of measurement. Patients were asked not to use their medication 12 hours before visits.

### Measurement of Serum Hyaluronan

Plasma was isolated from EDTA anticoagulated fasting blood samples and stored at −80°C until measurement. Serum hyaluronan was determined by enzyme-linked binding protein assay (Corgenix, Inc., Broomfield, Co, USA).

### Lung Function Measurement and Asthma Control Evaluation

Lung function was measured by means of electronic spirometer (PDD-301/s, Piston, Budapest, Hungary) according to the American Thoracic Society guidelines [38]. Three technically acceptable maneuvers were performed and the best was used. Forced expiratory volume in one second (FEV1), peak expiratory flow rate (PEF), and airway resistance (Raw) were measured. Asthma control was assessed using the Asthma Control Test (ACT) recommended by the current guideline [1].

### Statistics

Statistical analysis was performed using Graph Pad Prism software 5 (GraphPad Software, La Jolla, CA, USA), correction for age was made by SPSS Statistics V21 (International Business Machines Corporation, NY, USA). Data distribution was analyzed by D’Agostino-Pearson normality test. Data are expressed as median [interquartile range] except for the normally distributed values of age which are presented as the mean with standard deviation (SD). We used unpaired Student’s *t*-test for comparisons between the study groups with Welch correction for the adjustment for age. In case of not normally distributed data, Mann–Whitney *U*-test was performed. In the case of HA, the adjustment for age was done with Quade’s rank analysis of covariance. Correlations between HA and lung function parameters were determined with Spearman rank correlation. To adjust correlation between HA and Raw and between HA and ACT for patients’ age, partial rank correlation was used. AUC values of Receiver-Operating Characteristics (ROC) were carried out using standard methods and data are presented as AUC ROC (95% CI). p values <0.05 were considered significant in each calculation.

## Results

### Clinical Characteristics

Median age of asthmatic patients (N = 52) was 35 [29–43.75] years, whereas FEV1 (% of predicted) 92 [82.5–102], PEF (% of predicted) 90 [75.25–100], and Raw (% of predicted) 127 [102–165]. ACT total score of 21 [18]–[24] showed an acceptable level of disease control resulted by a 450 [0–500] µg daily dose of ICS (beclomethasone equivalent) used by the patients. Clinical data and inflammatory parameters of ANP (N = 36) and AP (N = 16) groups are summarized in Table 1. 27 non-pregnant and 12 pregnant patients received ICS treatment. The mean age of participants was higher in the ANP group compared to the AP group (41.28 (±14.3) vs. 31.13 (±5.123), respectively, p = 0.0005). Sampling was performed in the second or third trimester of gestation in all pregnant women (delivery data of 5 AP patients were not available). No difference was detected either in asthma severity or control or in daily dose of inhaled corticosteroids between the ANP and AP groups (Table 1).

**Table 1:** Clinical data and circulating hyaluronan levels of the four study groups.

	ANP (n = 36)	AP (n = 16)
Age (years)	41.28 (±14.3)	31.13 (±5.123)
Gestational age at sampling (weeks)	NA	23.5[17–32.5]
Gestational age at delivery (weeks)	NA	38 [38]–[39] ^n = 11^
Fetal birth weight (grams)	NA	3320 [3000–4000]^n = 11^
FEV_1_ (% of predicted)	90.5 [82.5–101.5]	94 [82.75–107]
PEF (% of predicted)	94.5 [76.75–100]	87.5 [71.25–96.75]
R_aw_ (% of predicted)	147 [102.3–168]	111 [98–129]^n = 15^
ACT total score	21.5 [18]–[24] ^n = 34^	21 [19]–[25] ^n = 15^
Daily dose of ICS (beclomethasone equivalent, µg)	500 [0–500]	225 [0–500]
Hyaluronan (HA; ng/mL)	37.4 [30.1–66.55]	27 [24.7–31.55]

ANP – asthmatic non-pregnant; AP – asthmatic pregnant; FEV_1_– forced expiratory volume in 1 second; PEF – peak expiratory flow rate; R_aw_ – airway resistance; ACT – Asthma Control Test; ICS – inhaled corticosteroids; NA-not applicable. All p values>0.05 except for age where p = 0.0005.

### Comparison of HA Levels between the Two Groups and its Relationship to Clinical Parameters

**Figure 1:**
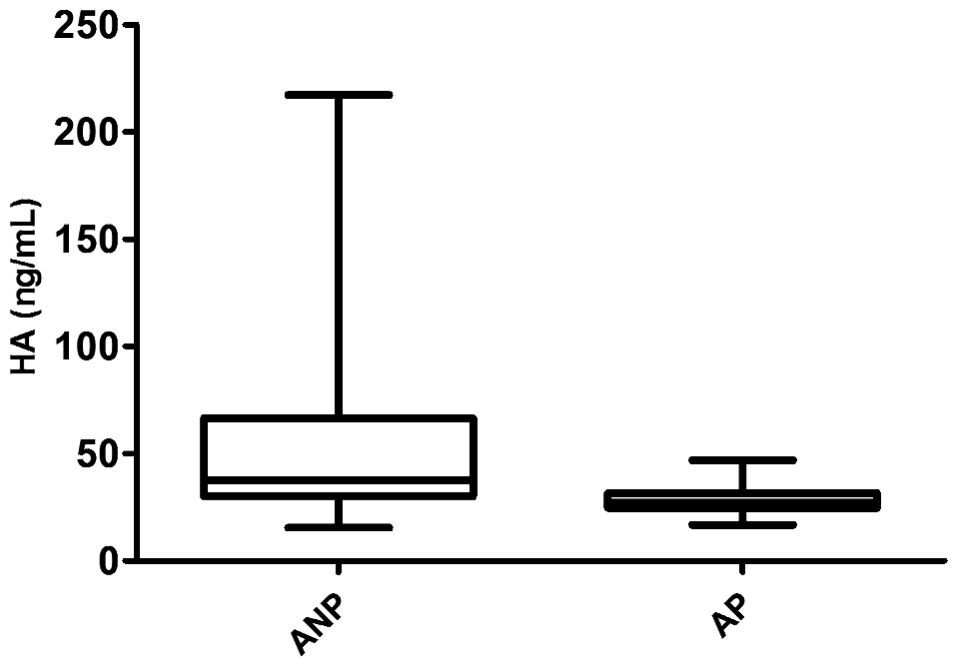
Circulating HA levels measured in asthmatic pregnant and non-pregnant women*. HA – hyaluronic acid; ANP – asthmatic non-pregnant; AP – asthmatic pregnant; p = 0.006; *data not adjusted for age.

Median circulating HA level in all asthmatic patients (N = 52) was 34.8 [25.6–46.48] ng/mL, and was related to age (p = 0.0064; r = 0.37). HA values were lower in AP than in ANP subjects (27 [24.7–31.55] vs. 37.4 [30.1–66.55] ng/mL, p = 0.006). After adjusting for age, significance changed to a trend (p = 0.056) (Figure 1).

**Figure 2:**
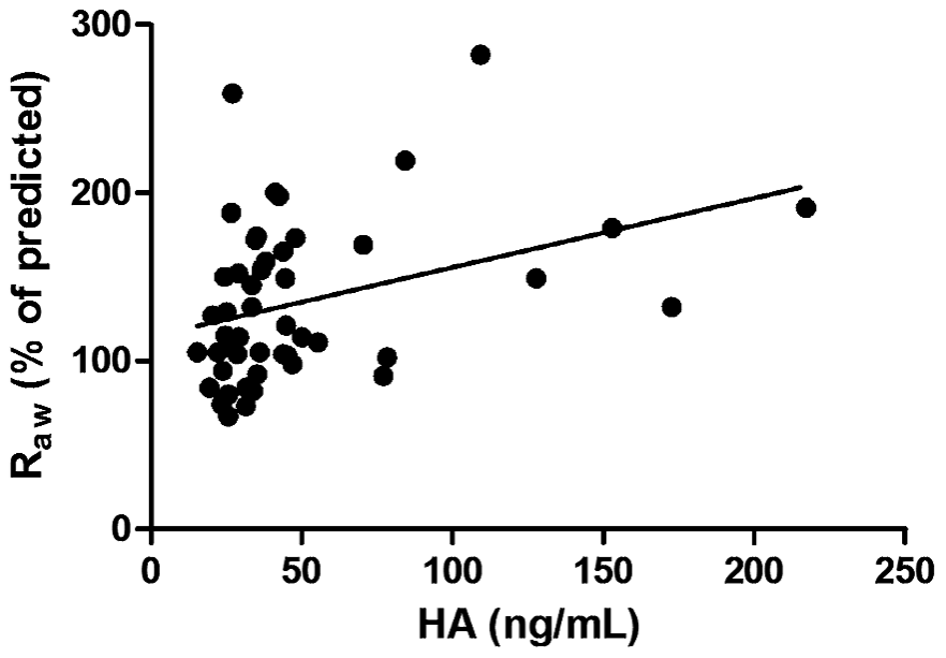
Correlation between HA levels and airway resistance in the whole asthmatic cohort. HA – hyaluronic acid; R_aw_ – Airway Resistance; p = 0.0055; r = 0.38.

A significant correlation was revealed between HA levels and Raw (p = 0.0055; r = 0.38; Figure 2) in the whole asthmatic cohort which remained significant after the adjustment for age (r = 0.326, p = 0.021); however no relationship was detected between HA levels and ACT total score, FEV1 or PEF. Circulating HA was not associated with the daily dose of inhaled corticosteroids.

**Figure 3:**
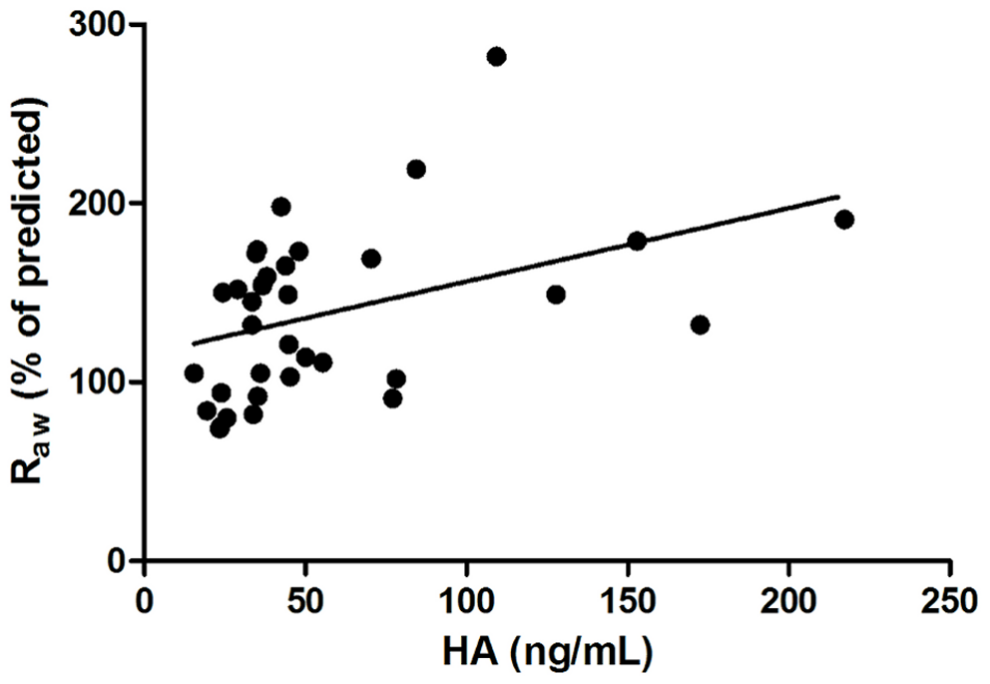
Correlation between HA levels and airway resistance in the asthmatic non-pregnant group. HA – hyaluronic acid; Raw – Airway Resistance; p = 0.004; r = 0.467.

**Figure 4:**
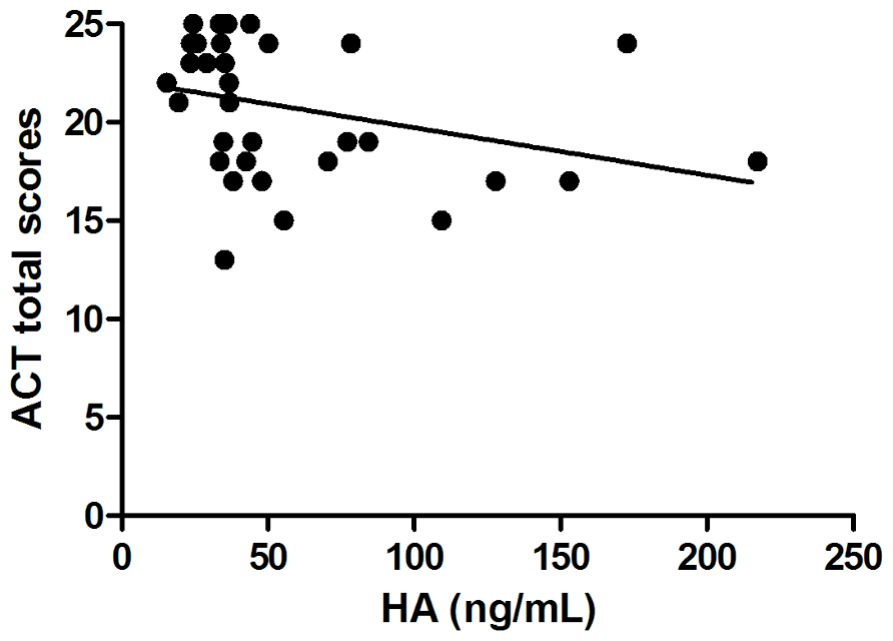
Negative correlation between HA levels and ACT total scores in the asthmatic non-pregnant group. HA – hyaluronic acid; ACT – Asthma Control Test; p = 0.01; r = −0.437.

In ANP group circulating HA levels were associated with Raw values (p = 0.004, r = 0.467; Figure 3) and age as well (p = 0.019, r = 0.39). In the same group an inverse correlation was found between serum HA levels and ACT total score (p = 0.01, r = −0.437) (Figure 4). After adjustment for age the association between HA and Raw (p = 0.014, r = 0.412) as well as between HA and ACT (p = 0.04, r = −0,36) were still present.

In AP patients no major neonatal or maternal complications were revealed. No correlation was detected between HA and clinical parameters of asthma or neonatal birth weight. There was no difference between HA levels of women in the second and the third trimester (p = 0.27).

### ROC Analysis of HA Values in Controlled and Uncontrolled Asthma

According to current asthma guidelines ACT total score ≥20 is the main determinant of well-controlled asthma. The potential value of HA in the determination of asthma control was analyzed using ROC analysis. ROC analyses of HA data were performed in subgroups of AP, ANP patients and also of all asthmatic patients with ACT total score above (controlled) and below (uncontrolled) 20. Statistical significance was proved in the case of HA with ACT score above and below 20 in the ANP group and in the whole asthmatic cohort.

**Figure 5:**
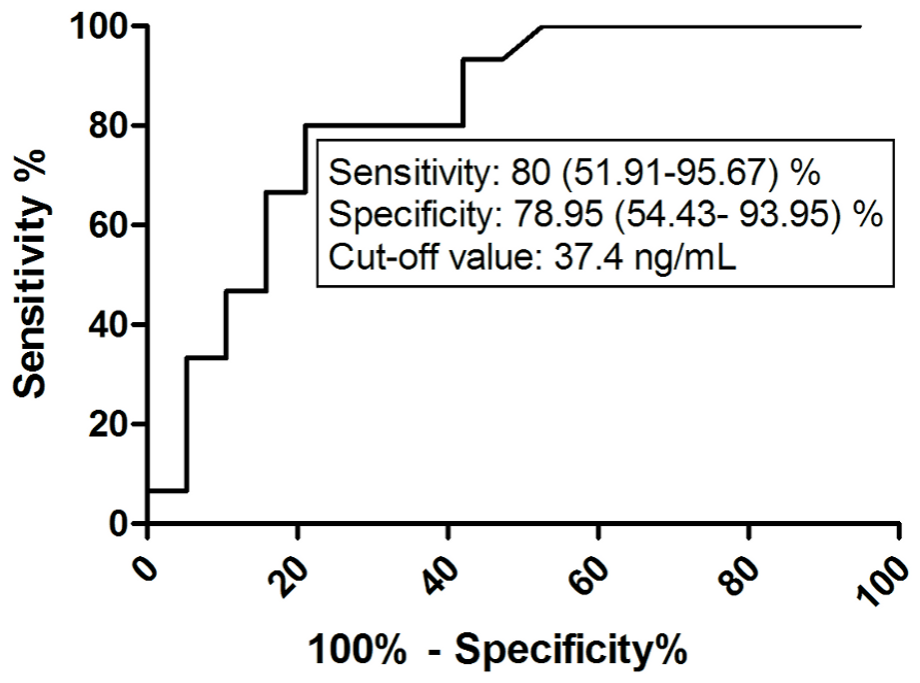
ROC analysis of HA values in asthmatic non-pregnant patients with controlled and uncontrolled disease according to ACT total score. HA – hyaluronic acid; ACT – asthma control test.

The area under the receiver operating characteristics curve for predicting asthma control was 0.826 (95% CI: 0.686–0.966) in the ANP group. The cut-off value of HA to discriminate between ANP patients with an ACT score above and below 20 was 37.4 ng/mL (sensitivity% (95% CI): 80 (51.91–95.67), specificity% (95% CI): 78.95 (54.43–93.95); p = 0.001) (Figure 5).

**Figure 6:**
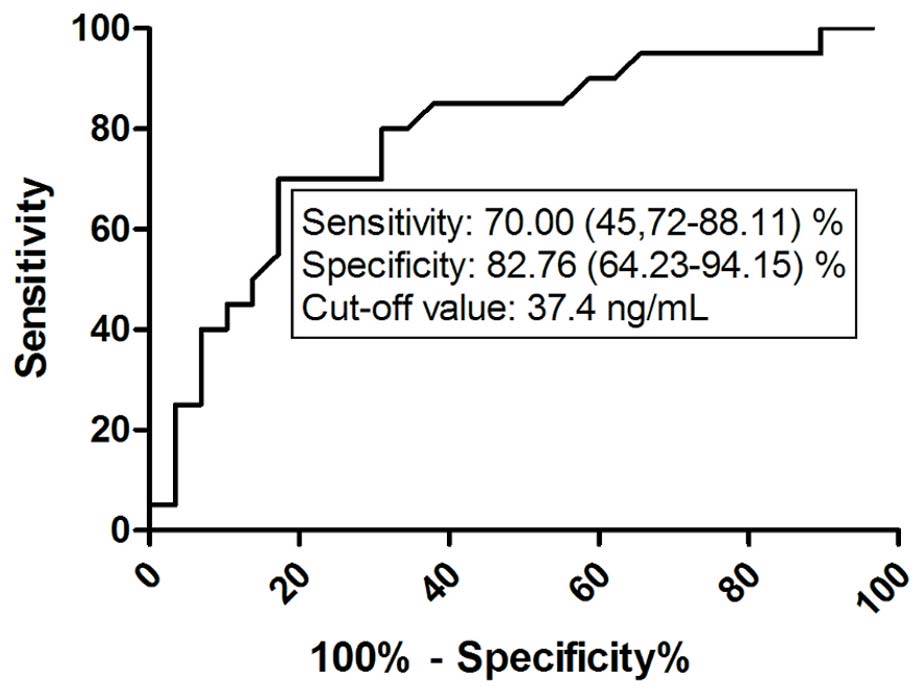
ROC analysis of HA values in the whole asthmatic cohort with controlled and uncontrolled disease according to ACT total score. HA – hyaluronic acid; ACT – asthma control test.

ROC analysis of HA values in the whole asthmatic patient group with Asthma Control Test total score above and below 20 yielded an AUC of 0.78 (95% CI: 0.65–0.92) with the 37.4 ng/mL cut-off value of HA to discriminate between patients with controlled and not controlled asthma (p = 0.0009, sensitivity% (95% CI): 70.00 (45.72–88.11), specificity% (95% CI): 82.76 (64.23–94.15) (Figure 6).

## Discussion

The aim of this study was to assess serum HA levels in asthmatic non-pregnant and asthmatic pregnant patients, and to reveal the possible relationship between HA and asthma control determinants in both groups. To our knowledge, this study was the first to investigate circulating HA values in asthmatic pregnancy.

The normal circulating concentration of HA may vary from 10 to 100 µg/L [27], and it correlates with age; however the average level of middle aged persons is between 30–40 µg/L [39]. In line with this, our data show a median circulating HA level of 34.8 µg/L in treated, mostly controlled asthmatics and support the correlation of circulating HA levels with age also in asthma. However one weakness of this study is the difference between the age in pregnant and non-pregnant groups.

Interestingly, lower level of circulating HA was found in asthmatic pregnant women compared to asthmatic non-pregnant patients in this study. Although the difference failed to reach statistical significance after correction for age, a trend still remained. Despite the well-known role of HA in morphogenesis, data on serum HA during pregnancy are not fully concordant. Kobayashi et al. observed increased serum HA levels in the third trimester of human pregnancy, especially close to term and a further increase was seen in labor suggesting an association with cervical ripening [35]. Elevated HA levels were also seen during pregnancy complicated with preeclampsia [36]. In our study HA levels were lower in pregnant than non-pregnant treated asthmatic patients. Asthma is known to be associated with systemic inflammation related to lung function and clinical symptoms [1]. Pregnancy on the other hand is characterized by immune tolerance resulting in attenuation of immunological responses [18]. Considerable amount of data supports that impaired maternal tolerance is responsible for adverse neonatal outcomes in gestations complicated with uncontrolled asthma, and that restored immune tolerance may help to maintain uncomplicated gestation in asthmatic women [40]. Therefore, it may be speculated that decrease in circulating HA level in pregnant group in this study (regardless of the presence of treated, mostly controlled asthma) was partially resulted by the immune tolerance characterizing pregnancy. The other cause may be pregnancy induced hemodilution [41]. However, lack of healthy pregnant and non-pregnant control values is a limitation of our study, though normal circulating HA levels of healthy non-pregnant people are known [42].

During tissue injury, repair, fibrosis and inflammation, HA levels elevate both in serum and tissue fluids [43]. Serum HA concentrations were shown to be comparable in small studies done on asthmatic and wheezing children [44] and asthmatic and healthy adults [45]; however it must be noted that available data on circulating HA concentrations in asthma are scarce. While the absence of healthy controls is a limitation of our study, it can be noted that our results show HA levels comparable to published normal values of subjects of similar age [39]. On the other hand, although HA values were within the normal range, they were positively associated to airway resistance and negatively related to asthma control. HA is involved in asthmatic inflammation in many ways including the regulation of airway remodeling which is characterized by airway muscle hyperplasia, increased mucus production and subepithelial fibrosis thus leading to thickened airway walls and narrowed airway calibers [46]. Airway resistance is an important tool in both the diagnosis and management of asthma, as it is an indicator of airway hyperresponsiveness and may reflect the airway remodeling process. Hence, based on the relationship between circulating HA level and Raw, serum HA assessment may help to estimate airway remodeling in asthma.

The potential value of circulating HA measurement in the determination of asthma control was analyzed using ROC analysis. We found that a cut-off level of 37.4 ng/mL is suitable to discriminate with acceptable sensitivity and specificity between patients suffering from well-controlled or uncontrolled asthma and thus help to identify patients with impaired asthma control; however the diagnostic yield of HA for the detection of uncontrolled asthma was much lower in asthmatic pregnancy.

In summary, circulating HA seems to be a marker of disease control in asthma, as it correlates with airway resistance and effectively discriminates between well-controlled and uncontrolled asthma. The best utility of HA serving as a screening tool in the evaluation of asthma control was detected in non-pregnant patients. Decreased level of HA in asthmatic pregnant women is presumably the result of pregnancy induced immune tolerance and attenuated systemic inflammatory responses which weaken the probable value of serum HA as a marker of asthma control during pregnancy.

## References

[1] Global Initiative for Asthma. Available: http://www.ginasthma.org. Accessed 2013 Sep 17.

[2] Rabe KF, Adachi M, Lai CK, Soriano JB, Vermeire PA, et al (2004) Worldwide severity and control of asthma in children and adults: the global asthma insights and reality surveys. J Allergy Clin Immunol 114(1): 40–7. 15241342 10.1016/j.jaci.2004.04.042

[3] Louis R, Lau LC, Bron AO, Roldaan AC, Radermecker M, et al (2000) The relationship between airways inflammation and asthma severity. Am J Respir Crit Care Med 161: 9–16. 10619791 10.1164/ajrccm.161.1.9802048

[4] Sont JK, Han J, van Krieken JM, Evertse CE, Hooijer R, et al (1996) Relationship between the inflammatory infiltrate in bronchial biopsy specimens and clinical severity of asthma in patients treated with inhaled steroids. Thorax 51: 496–502. 8711677 10.1136/thx.51.5.496 PMC473594

[5] Volbeda F, Broekema M, Lodewijk ME, Hylkema MN, Reddel HK, et al (2013) Clinical control of asthma associates with measures of airway inflammation. Thorax 68(1): 19–24. 23042704 10.1136/thoraxjnl-2012-201861

[6] Yokoyama A, Kohno N, Fujino S, Hamada H, Inoue Y, et al (1995) Circulating interleukin-6 levels in patients with bronchial asthma. Am J Respir Crit Care Med 151(5): 1354–1358. 7735584 10.1164/ajrccm.151.5.7735584

[7] Silvestri M, Bontempelli M, Giacomelli M, Malerba M, Rossi GA, et al (2006) High serum levels of tumour necrosis factor-alpha and interleukin-8 in severe asthma: markers of systemic inflammation? Clin Exp Allergy 36(11): 1373–81. 17083347 10.1111/j.1365-2222.2006.02502.x

[8] Mukhopadhyay S, Hoidal JR, Mukherjee TK (2006) Role of TNFalpha in pulmonary pathophysiology. Respir Res 11 7: 125. 10.1186/1465-9921-7-125 PMC1613248 17034639

[9] Olafsdottir IS, Gislason T, Thjodleifsson B, Olafsson I, Gislason D, et al (2005) C reactive protein levels are increased in non-allergic but not allergic asthma: a multicentre epidemiological study. Thorax 60(6): 451–454. 15923243 10.1136/thx.2004.035774 PMC1747429

[10] Wood LG, Baines KJ, Fu J, Scott HA, Gibson PG (2012) The neutrophilic inflammatory phenotype is associated with systemic inflammation in asthma. Chest 142(1): 86–93. 22345378 10.1378/chest.11-1838

[11] Takemura M, Matsumoto H, Niimi A, Ueda T, Matsuoka H, et al (2006) High sensitivity C-reactive protein in asthma. Eur Respir J 27(5): 908–912. 16707391 10.1183/09031936.06.00114405

[12] Ivancsó I, Toldi G, Bohács A, Eszes N, Müller V, et al (2013) Relationship of circulating soluble urokinase plasminogen activator receptor (suPAR) levels to disease control in asthma and asthmatic pregnancy. PLoS One. 8(4): e60697. 10.1371/journal.pone.0060697 PMC3614899 23565268

[13] Kwon HL, Belanger K, Bracken MB (2003) Asthma prevalence among pregnant and childbearing-aged women in the United States: estimates from national health surveys. Ann Epidemiol 13: 317–324. 12821270 10.1016/s1047-2797(03)00008-5

[14] Demissie K, Breckenridge MB, Rhoads GG (1998) Infant and maternal outcomes in the pregnancies of asthmatic women. Am J Respir Crit Care Med 158: 1091–1095. 9769265 10.1164/ajrccm.158.4.9802053

[15] Breton MC, Beauchesne MF, Lemière C, Rey E, Forget A, et al (2009) Risk of perinatal mortality associated with asthma during pregnancy. Thorax 64: 101–106. 19008298 10.1136/thx.2008.102970

[16] Schatz M (1999) Interrelationships between asthma and pregnancy: a literature review. J Allergy Clin Immunol 103: S330–336. 9949333 10.1016/s0091-6749(99)70258-7

[17] Murphy VE, Clifton VL, Gibson PG (2006) Asthma exacerbations during pregnancy: incidence and association with adverse pregnancy outcomes. Thorax 61(2): 169–76. 16443708 10.1136/thx.2005.049718 PMC2104591

[18] Tamasi L, Horvath I, Bohacs A, Muller V, Losonczy GY, et al (2011) Asthma in pregnancy - Immunological changes and clinical management. Respir Med 105: 159–164. 21145223 10.1016/j.rmed.2010.11.006

[19] Murphy VE, Gibson PG (2011) Asthma in pregnancy. Clin Chest Med 32(1): 93–110, ix. 10.1016/j.ccm.2010.10.001 21277452

[20] Schatz M, Dombrowski MP, Wise R, Lai Y, Landon M, et al (2010) The relationship of asthma-specific quality of life during pregnancy to subsequent asthma and perinatal morbidity. J Asthma 47: 46–50. 20100020 10.3109/02770900903483758 PMC3249656

[21] Almond A (2007) Hyaluronan. Cell Mol Life Sci 64: 1591–1596. 17502996 10.1007/s00018-007-7032-z PMC11136421

[22] Olczyk P, Komosinska-Vassev K, Winsz-Szczotka K, Kuznik-Trocha K, Olczyk K (2008) [Hyaluronan: structure, metabolism, functions, and role in wound healing]. Postepy Hig Med Dosw 62: 651–659. 19057507

[23] Grootveld MC, Henderson EB, Farrell A, Blake R, Parkes HG, et al (1991) Oxidative damage to hyaluronate and glucose in synovial fluid during exercise of the inflamed joint: detection of abnormal low molecular weight metabolites by proton nuclear magnetic spectroscopy. Biochem J 273 (pt 2): 459–467. 10.1042/bj2730459 PMC1149867 1991041

[24] Gao F, Koenitzer JR, Tobolewski JM, Jiang D, Liang J, et al (2008) Extracellular superoxide dismutase inhibits inflammation by preventing oxidative fragmentation of hyaluronan. J Biol Chem 283: 6058–6066. 18165226 10.1074/jbc.M709273200 PMC2268976

[25] Jiang D, Liang J, Noble PW (2007) Hyaluronan in tissue injury and repair. Annu Rev Cell Dev Biol 23: 435–61. 17506690 10.1146/annurev.cellbio.23.090506.123337

[26] Stern R, Asari AA, Sugahara KN (2006) Hyaluronan fragments: an information-rich system. Eur J Cell Biol. 85(8): 699–715. 10.1016/j.ejcb.2006.05.009 16822580

[27] Laurent TC (1987) Biochemistry of Hyaluronan. Acta Octolaryngol Suppl 442: 7–24. 10.3109/00016488709102833 3124495

[28] Jiang D, Liang J, Noble PW (2011) Hyaluronan as an immune regulator in human diseases. Physiol Rev. 91(1): 221–264. 10.1152/physrev.00052.2009 PMC3051404 21248167

[29] Söderberg M, Bjermer L, Hällgren R, Lundgren R (1989) Increased hyaluronan (hyaluronic acid) levels in bronchoalveolar lavage fluid after histamine inhalation. Int Arch Allergy Appl Immunol 88(4): 373–376. 2722257 10.1159/000234719

[30] Vignola AM, Chanez P, Campbell AM, Souques F, Lebel B, et al (1998) Airway inflammation in mild intermittent and in persistent asthma. Am J Respir Crit Care Med 157(2): 403–409. 9476850 10.1164/ajrccm.157.2.96-08040

[31] Bousquet J, Chanez P, Lacoste JY, Enander I, Venge P, et al (1991) Indirect evidence of bronchial inflammation assessed by titration of inflammatory mediators in BAL fluid of patients with asthma. J Allergy Clin Immunol 88: 649–660. 1918730 10.1016/0091-6749(91)90159-l

[32] Liang J, Jiang D, Jung Y, Xie T, Ingram J, et al (2011) Role of hyaluronan and hyaluronan-binding proteins in human asthma. J Allergy Clin Immunol 128(2): 403–411.e3. 21570715 10.1016/j.jaci.2011.04.006 PMC3149736

[33] Ohkawara Y, Tamura G, Iwasaki T, Tanaka A, Kikuchi T, et al (2000) Activation and transforming growth factor-beta production in eosinophils by hyaluronan. Am J Respir Cell Mol Biol 23: 444–451. 11017908 10.1165/ajrcmb.23.4.3875

[34] Cordo-Russo R, Garcia MG, Barrientos G, Orsal AS, Viola M, et al (2009) Murine abortion is associated with enhanced hyaluronan expression and abnormal localization at the fetomaternal interface. Placenta. 30(1): 88–95. 10.1016/j.placenta.2008.10.013 19059644

[35] Kobayashi H, Sun GW, Tanaka Y, Kondo T, Terao T (1999) Serum hyaluronic acid levels during pregnancy and labor. Obstet Gynecol 93(4): 480–484. 10214818 10.1016/s0029-7844(98)00526-2

[36] Uzun H, Konukoglu D, Albayrak M, Benian A, Madazli R, et al (2010) Increased maternal serum and cord blood fibronectin concentrations in preeclampsia are associated with higher placental hyaluronic acid and hydroxyproline content. Hypertens Pregnancy 29(2): 153–162. 20367505 10.3109/10641950902968619

[37] Berg S, Engman A, Holmgren S, Lundahl T, Laurent TC (2001) Increased serum hyaluronan in severe preeclampsia and eclampsia. Scand J Clin Lab Invest 61: 131–138. 11347980 10.1080/00365510151097647

[38] Miller MR, Hankinson J, Brusasco V, Burgos F, Casaburi R, et al (2005) Standardisation of spirometry. Eur Respir J 26: 319–338. 16055882 10.1183/09031936.05.00034805

[39] Lindqvist U, Laurent TC (1992) Serum hyaluronan and aminoterminal propeptide of type III procollagen. Variation with age. Scand J Clin Lab Invest 1992 52: 613–621. 10.3109/00365519209115504 1455153

[40] Tamási L, Bohács A, Pállinger E, Falus A, Rigó J Jr, et al (2005) Increased interferon-gamma- and interleukin-4-synthesizing subsets of circulating T lymphocytes in pregnant asthmatics. Clin Exp Allergy 35: 1197–1203. 16164448 10.1111/j.1365-2222.2005.02322.x

[41] Blackburn ST, Loper DL (1992) Maternal, fetal, and neonatal physiology. A clinical perspective. Philadelphia: W.B. Saunders Co.; p.160–162, 171, 202–203, 222–228.

[42] Engstrom-Laurent A, Laurent UBG, Lilia K, Laurent TC (1985) Concentration of sodium hyaluronate in serum. Scand J Clin Lab Invest 45: 497–504. 3906849 10.3109/00365518509155249

[43] Jiang D, Liang J, Noble PW (2007) Hyaluronan in Tissue Injury and Repair. Ann Rev Cell Dev Biol 23: 435–461. 17506690 10.1146/annurev.cellbio.23.090506.123337

[44] Fuji Y, Shima M, Ando M, Adachi M, Tsunetoshi Y (2002) Effect of air pollution and environmental tobacco smoke on serum hyaluronate concentrations in school children. Occup Environ Med 59: 124–128. 11850556 10.1136/oem.59.2.124 PMC1740261

[45] DaVeiga SP, Swaidani S, Comhair SA, Erzurum SC, Aronica MA (2009) Elevation of Hyaluronan Levels in Human Subjects with Atopic Asthma. 10.1164/ajrccm-conference. 179: A2852 (abstract)

[46] Boulet L, Belanger M, Carrier G (1995) Airway responsiveness and bronchial-wall thickness in asthma with or without fixed airflow obstruction. Am. J. Respir Crit Care Med 152: 865–871. 10.1164/ajrccm.152.3.7663797 7663797

